# Sharing raw data from clinical trials: what progress since we first asked “Whose data set is it anyway?”

**DOI:** 10.1186/s13063-016-1369-2

**Published:** 2016-05-04

**Authors:** Andrew J. Vickers

**Affiliations:** Department of Epidemiology and Biostatistics, Memorial Sloan Kettering Cancer Center, 485 Lexington Avenue, 2nd Floor, New York, NY 10017 USA

## Abstract

Ten years ago, one of the first papers published in *Trials* was a commentary entitled “Whose data set is it anyway?” The commentary pointed out that trialists routinely refused requests for data sharing and argued that this attitude was a community standard that had no rational basis. At the time, there had been few calls for clinical trial data sharing and certainly no institutional support. Today the situation could not be more different. Numerous organizations now recommend or require raw data to be made available, including the International Committee of Medical Journal Editors, which recently proposed that clinical trial data sharing be a “condition of … publication.” Furthermore, the literature is replete with papers covering an enormously wide variety of topics on data sharing. But despite a tectonic shift in attitudes, we are yet to see clinical trial data sharing become an unquestioned norm, where a researcher can readily download a data set from a trial almost as easily as they can now download a copy of the published paper. The battle over the next few years is to go beyond changing minds to ensuring that real data sets are routinely made available.

## Background

It is often said that people don’t change. Indeed, it is almost a point of academic pride to be cynical about our capacity for change, and to view optimists as naïve and callow. Yet change often happens remarkably quickly. In 2004, President George W Bush used opposition to gay marriage to motivate his supporters; in 2015, the Supreme Court legalized gay marriage with the majority support of the American public. In 1988, Jesse Jackson's run for president was considered a token exercise, leading *Newsweek* magazine to ask: “What makes Jesse run?”; 2008 saw the election of Barack Obama

I think we have seen similarly rapid change in terms of attitudes to data sharing. Ten years ago, one of the first papers published in *Trials* was a commentary entitled “Whose data set is it anyway?” [1]. The commentary pointed out that trialists tended to see trial data as their personal property and would routinely refuse requests for data sharing. As just one example, a National Institutes of Health (NIH) investigator refused to release data from the control group of a published trial, requested to help the sample size calculation for a new study. Anecdotes were complemented by survey data showing that three quarters of trialists, as well as pharmaceutical industry groups, were opposed to making raw data available after trial publication.

The key argument of the commentary was that this attitude was a community standard that had no rational basis. Arguments against data sharing were entirely trivial, such as spurious concerns about patient confidentiality – in most cases, it is straightforward to deidentify a data set – or complaints about the time and effort an investigator would have to invest in making a data set ready for sharing (would they not already have had to do so in order to analyze the data for publication?). Moreover, other disciplines, from genomic researchers to economists, routinely made data freely available. The clinical trialists did not share data because that is not what clinical trialists did, a social norm not much different in form from attitudes towards gays and blacks.

At the time of the 2006 *Trials* commentary, only a handful of papers had previously called for data sharing. There was a paper published 10 years previously in the *BMJ*, the title of which, in a case of inadvertent plagiarism, the commentary had mirrored [2]. Kirwan’s review of data-sharing attitudes [3] had been cited in the commentary and, of course, one could go all the way back to the first issue of *Biometrika*, in which Galton called for publication of data alongside the primary analyses [4]. It might also be noted that at the time of the commentary, no major institution had called for clinical trial data sharing to be a matter of course.

## Main text

Today, ten years on from publication of the *Trials* commentary, the situation could not be more different: numerous organizations now recommend or require raw data to be made available, and the literature is replete with papers covering an enormously wide variety of topics on data sharing. In terms of recommendations, clinical trial data sharing has been the subject of a report from the Institute of Medicine, [5, 6] which recommends, among other things, that funders should require trialists to share data and provide appropriate support to do so. Funders have certainly shown interest, with a group of 17 funders led by the Wellcome Trust publishing a “statement of purpose” on data sharing, including a set of principles [7]. Some funders have gone beyond principles: the National Health, Lung and Blood Institute [8], for instance, has developed specific data-sharing practices and a data repository currently including over half a million patients from over 100 trials and observational studies. In a recent, dramatic development, the International Committee of Medical Journal Editors has recommended that as a “condition of … publication” of a trial report, journals will “require authors to share with others the deidentified individual-patient data no later than 6 months after publication” [9]. If fully enacted, this recommendation would transform the landscape of clinical trial data sharing. The *BMJ* has already taken the lead, with a policy that now requires data sharing “on request” for all trials [10]. Some pharmaceutical groups are following suit, with Roche stating that they will provide individual patient data from clinical trials in response to requests with “good scientific merit” [11]. Project Data Sphere [12] is an industry-led initiative to provide a software platform for clinical trial data sharing, and initiatives by GSK and Medtronic to share clinical trial data have received wide praise [10].

Alongside these initiatives and recommendations, a substantial literature has been published that investigates data sharing as a research topic. We have seen papers developing data standards for clinical trials in narrow fields (for instance, polycystic kidney disease [13] and spinal cord injury [14]); technical papers on deidentification [15]; numerous surveys about the practice of or attitudes to data sharing [16–21]; discussion of ethical issues [22] (including those pertaining to highly localized issues in countries such as South Africa [23] or Vietnam [24]); and practical guidance on how to share data [25, 26].

All that said, the war is far from won: attitudes have shifted dramatically, tectonically, but we are yet to see clinical trial data sharing become an unquestioned norm, where, say, a researcher can readily download a data set from a trial almost as easily as they can now download the trial publication. And there are still battles to be fought: the Pharmaceutical Research and Manufacturers of America, for instance, claims to be “firmly committed to enhancing public health” but current guidelines on communication of trial results [27] speak of making clinical trial data accessible only to investigators.

## Conclusions

I draw three conclusions from my experiences in promoting clinical trial data sharing. First, we are blessed to be working in a discipline in which reason matters, and where individuals will change their attitudes when presented with sound arguments. Second, dramatic cultural change is indeed possible within a short period of time, if the cause is just. Third, changing attitudes is not enough. In the “states of change” model describing how, say, a smoker quits smoking cigarettes, “contemplation” and “preparation” need to be followed by “action” and “maintenance.” The 2006 commentary ended: “Let’s make sharing of raw data a commonplace, natural part of the clinical trials process, in the same way that we view obtaining ethical approval or publication of the trial results.” Our job over the next decades will be to make sure, first, that this does indeed happen and, second, that it stays that way.

## References

[1] Vickers AJ (2006). Whose data set is it anyway? Sharing raw data from randomized trials. Trials.

[2] Delamothe T (1996). Whose data are they anyway?. BMJ (Clinical research ed).

[3] Kirwan JR (1997). Making original data from clinical studies available for alternative analysis. J Rheumatol.

[4] Galton F (1901). Biometry. Biometrika.

[5] Lo B (2015). Sharing clinical trial data: maximizing benefits, minimizing risk. JAMA.

[6] Committee on Strategies for Responsible Sharing of Clinical Trial Data, Board on Health Sciences Policy, Institute of Medicine (2014). The National Academies Collection: Reports funded by National Institutes of Health. Discussion framework for clinical trial data aharing: guiding principles, elements, and activities.

[7] Vickers AJ (2011). Making raw data more widely available. BMJ (Clinical research ed).

[8] Coady SA, Wagner E (2013). Sharing individual level data from observational studies and clinical trials: a perspective from NHLBI. Trials.

[9] Taichman DB, Backus J, Baethge C, Bauchner H, de Leeuw PW, Drazen JM (2016). Sharing clinical trial data – A proposal from the International Committee of Medical Journal Editors. N Engl J Med.

[10] Loder E, Groves T (2015). The BMJ requires data sharing on request for all trials. BMJ (Clinical research ed).

[11] Our commitment to data sharing. http://www.roche.com/research_and_development/who_we_are_how_we_work/clinical_trials/our_commitment_to_data_sharing.htm?acc1=tab11. Accessed 30 Apr 2016.

[12] Project Data Sphere. www.projectdatasphere.org. Accessed 30 Apr 2016.

[13] Perrone RD, Neville J, Chapman AB, Gitomer BY, Miskulin DC, Torres VE (2015). Therapeutic area data standards for autosomal dominant polycystic kidney disease: a report from the polycystic kidney disease outcomes consortium (PKDOC). Am J Kidney Dis.

[14] Biering-Sorensen F, Alai S, Anderson K, Charlifue S, Chen Y, DeVivo M (2015). Common data elements for spinal cord injury clinical research: a National Institute for Neurological Disorders and Stroke project. Spinal Cord.

[15] Dankar FK, El Emam K, Neisa A, Roffey T (2012). Estimating the re-identification risk of clinical data sets. BMC Med Inform Decis Mak.

[16] Rathi V, Dzara K, Gross CP, Hrynaszkiewicz I, Joffe S, Krumholz HM (2012). Sharing of clinical trial data among trialists: a cross sectional survey. BMJ (Clinical research ed).

[17] Rathi VK, Strait KM, Gross CP, Hrynaszkiewicz I, Joffe S, Krumholz HM (2014). Predictors of clinical trial data sharing: exploratory analysis of a cross-sectional survey. Trials.

[18] Hopkins C, Sydes M, Murray G, Woolfall K, Clarke M, Williamson P (2016). UK publicly funded Clinical Trials Units supported a controlled access approach to share individual participant data but highlighted concerns. J Clin Epidemiol.

[19] Tudur Smith C, Dwan K, Altman DG, Clarke M, Riley R, Williamson PR (2014). Sharing individual participant data from clinical trials: an opinion survey regarding the establishment of a central repository. PLoS One.

[20] Alsheikh-Ali AA, Qureshi W, Al-Mallah MH, Ioannidis JP (2011). Public availability of published research data in high-impact journals. PLoS ONE.

[21] Savage CJ, Vickers AJ (2009). Empirical study of data sharing by authors publishing in PLoS journals. PLoS One.

[22] Mello MM, Francer JK, Wilenzick M, Teden P, Bierer BE, Barnes M (2013). Preparing for responsible sharing of clinical trial data. N Engl J Med.

[23] Denny SG, Silaigwana B, Wassenaar D, Bull S, Parker M (2015). Developing ethical practices for public health research data sharing in South Africa: the views and experiences from a diverse sample of research stakeholders. J Empir Res Hum Res Ethics.

[24] Merson L, Phong TV, le Nhan NT, Dung NT, Ngan TT, Kinh NV (2015). Trust, respect, and reciprocity: informing culturally appropriate data-sharing practice in Vietnam. J Empir Res Hum Res Ethics.

[25] Tudur Smith C, Hopkins C, Sydes MR, Woolfall K, Clarke M, Murray G (2015). How should individual participant data (IPD) from publicly funded clinical trials be shared?. BMC Med.

[26] Hrynaszkiewicz I, Norton ML, Vickers AJ, Altman DG (2010). Preparing raw clinical data for publication: guidance for journal editors, authors, and peer reviewers. Trials.

[27] Principles on conduct of clinical trials: communication of clinical trial results. http://phrma.org/sites/default/files/pdf/042009_clinical_trial_principles_final_0.pdf. Accessed April 30th, 2016..

